# Prediction of cerebral palsy and cognitive delay among high‐risk children in a developing nation: A successful early detection programme

**DOI:** 10.1111/dmcn.16197

**Published:** 2024-12-28

**Authors:** Gemunu Hewawitharana, Nuwan Darshana ILA, Asha Madhushani UI, Sadeepi Chathuranga DP, Nirosha Priyangika DI, Bimba Hewawitharana, Champa Wijesinghe, Piyadasa Kodituwakku, John Phillips, RG Yashoda Madumadhavie, RD Susantha Kumara, DJ Danunga Mihiran, KV. Kanchana Nilukshika

**Affiliations:** ^1^ Paediatric Neurology Unit Teaching Hospital Galle Sri Lanka; ^2^ Department of Paediatric Neurology Ninewells Hospital, NHS Tayside, and University of Dundee UK; ^3^ Department of Community Medicine, Faculty of Medicine University of Ruhuna Galle Sri Lanka; ^4^ Department of Pediatrics University of New Mexico School of Medicine Albuquerque New Mexico USA

## Abstract

**Aim:**

To determine the feasibility of combining the Hammersmith Infant Neurological Examination (HINE) and General Movements Assessment (GMA) within a standard follow‐up schedule to predict developmental outcomes in infants at risk in low‐ and middle‐income countries (LMICs).

**Method:**

A total of 201 Sri Lankan infants (128 male, 73 female) were prospectively assessed with the GMA before 44 weeks (writhing movements) and at 3 to 4 months (fidgeting movements), followed by the HINE at 5 to 6 months. Developmental outcomes were assessed using the Bayley Scales of Infant and Toddler Development, Fourth Edition and clinical assessment after 24 months.

**Results:**

The sensitivity of predicting cerebral palsy (CP) was lower with a single GMA assessment (writhing 89.5%, fidgeting 94.7%) or HINE (89.5%) compared to all three assessments combined (sensitivity 100%, 95% confidence interval [CI] = 82.4–100.0). The GMA and HINE were less predictive of non‐CP‐related developmental delays, particularly when single assessments were used (< 65% for all domains) compared to all three assessments combined (motor sensitivity > 86.9%, 95% CI = 66.4–97.2; cognitive sensitivity > 86.7%, 95% CI = 69.3–96.2; social–emotional sensitivity > 83.3%, 95% CI = 65.3–94.4). Specificity was lower for the prediction of CP‐related (40.1%) and non‐CP‐related developmental delays (< 46.0% for all).

**Interpretation:**

In an LMIC such as Sri Lanka, with limited access to specialist care and neuroimaging, combining two GMA measures and the HINE identified most infants with CP‐related and non‐CP‐related developmental delay, thereby allowing targeted early intervention therapies.

## Graphical abstract

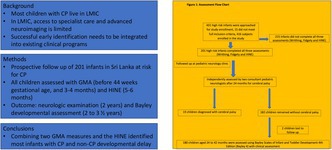

201 infants at risk for developmental disorders were assessed using two General Movements Assessments (GMA) and one Hammersmith Infant Neurological Examination (HINE), integrated into routine follow up care. Developmental outcome was assessed with a neurologic examination at 2 years, and the Bayley Scales of Infant and Toddler Development at 2 ‐ 3.5 years of age. Using a combination of GMA measures and the HINE, CP was predicted with 100% sensitivity, and non‐CP delays were predicted with 83%‐87% sensitivity. Although specificity was lower, this approach provides a practical method of identifying most infants who will benefit from early intervention therapy, even in countries such as Sri Lanka where access to neuroimaging and specialist care is limited..

Plain language summary: https://onlinelibrary.wiley.com/doi/10.1111/dmcn.16236

**This original article is commented by dos Santos on pages** 829–830 **of this issue.**

**Abbreviations**
AUC: area under the curve
Bayley‐4: Bayley Scales of Infant and Toddler Development, Fourth Edition
GMA: General Movements Assessment
HIC: high‐income country
HINE: Hammersmith Infant Neurological Examination
LMIC: low‐ and middle‐income country
ROC: receiver operating characteristic

**What this paper adds**

- Successful prediction of cerebral palsy (CP) and other developmental delays without imaging data is possible in a developing nation.
- Integrating formal assessments into routine care improves the early identification of neurodevelopmental disorders in resource‐limited settings.
- Combining two General Movements Assessments (GMAs) with total Hammersmith Infant Neurological Examination (HINE) scores are highly predictive of CP.
- Combined GMA and HINE scores are also predictive of cognitive and social–emotional delays in children.

In high‐income Western countries, an extensive range of tools have been developed and recommendations proposed for the early detection of neurodevelopmental disorders, including cerebral palsy (CP), in at‐risk children.^1^, ^2^ The current recommendation for predicting CP in children with risk factors is to screen all children who are at risk for CP before 5 months of age using brain magnetic resonance imaging (MRI), in addition to performing either the Hammersmith Infant Neurological Examination (HINE)^3^ or the General Movements Assessment (GMA).^4^ Unfortunately, studies supporting the high predictive power of the GMA and HINE are from high‐income countries (HICs); only limited data are available from low‐ and middle‐income countries (LMICs) regarding the use of the GMA and HINE in predicting developmental disorders.

A practical issue in LMICs such as Sri Lanka is that access to advanced neuroimaging is limited; therefore, screening all at‐risk infants with MRI is not feasible. Furthermore, limited resources make it difficult to perform multiple assessments over time in all children at risk for developmental disabilities. Therefore, an approach to early diagnosis is needed for use in resource‐limited settings; this needs to be feasible and have reasonable predictive power for both motor abnormalities such as CP and cognitive delay. Without a practical approach to early diagnosis, it is not possible to appropriately target developmental therapy for children who will benefit most from early intervention.

We report results from a prospective study of early detection of CP and cognitive and social–emotional delay in infants with neonatal risk factors attending a Sri Lankan tertiary care facility. The GMA and HINE were integrated into the standard clinic follow‐up schedule to minimize burden on families, which we refer to as a ‘modified approach’ to early detection. Our goal was to determine the benefit of using a combination of two GMAs (one writhing movement [GMA 1] before discharge, one fidgeting movement [GMA 2] at a corrected age of 3–4 months) and the HINE at a corrected age of 5 to 6 months to predict later development of motor delay. In addition, because motor skills have a foundational role in the development of perceptual and cognitive skills,^5^ we also explored the utility of using these tests for the early identification of cognitive delay. Our hypothesis was that combining two GMAs and a single HINE into a standard follow‐up clinical schedule would provide a sensitive measure for the early detection of CP and other developmental delays.

## METHOD

### Study design and participants

This prospective, longitudinal study was conducted at the Paediatric Neurology Unit of Teaching Hospital Karapitiya, a tertiary care paediatric unit in the southern province of Sri Lanka. Participants were recruited between 1st June 2018 and 31st August 2020. There are more than 1000 births per month in its catchment area, with most infants receiving outpatient services through the hospital's paediatric units. Research participants were recruited as a consecutive sample of high‐risk infants registered at the Paediatric Neurology Unit.

Study participants were infants with risk factors for developing CP or developmental delay, including preterm birth or neonatal encephalopathy, such as neonatal hypoxia, neonatal seizures, or central nervous system infections (Table 1). Infants who had poor life expectancy because of known genetic abnormalities, neurodegenerative disorders such as spinal muscular atrophy, or severe congenital cardiac abnormalities were excluded. For families with transport problems, transport was provided for the follow‐up visits, when possible.

**TABLE 1:** Common risk factors identified among infants in the study cohort (*n* = 201).

Risk factor	Children with all three assessments (*n* = 201), *n* (%)^a^	Children without all three assessments (*n* = 215), *n* (%)^a^	*p* ^b^
Low birthweight	112 (55.7)	127 (59.1)	0.490
Preterm birth	105 (52.2)	120 (55.8)	0465
Birth asphyxia	26 (12.9)	30 (13.9)	0.764
CNS infections	28 (13.9)	28 (13.0)	0.787
Ventilated after birth	46 (22.9)	50 (23.2)	0.928
Symptomatic hypoglycaemia	20 (10.0)	14 (6.5)	0.200
Neonatal seizures	36 (17.9)	27 (12.5)	0.128
Neonatal resuscitation	52 (25.9)	34 (15.8)	0.114
Congenital infections	6 (3.0)	3 (1.4)	0.267

Abbreviation: CNS, central nervous system.

^a^ Many infants had multiple risk factors; therefore, percentages do not add up to 100%.

^b^ Compared using a Z‐test for two proportions. Risk factors were not statistically different in the two groups.

The sample size required to assess diagnostic test accuracy,^6^ with an expected sensitivity of 92% and specificity of 82%, was calculated using an estimated prevalence of developmental disorders of 17%, a power of 80%, and a precision of 15%. Based on these parameters, the sample sizes required to estimate sensitivity and specificity were calculated separately; the sample size for specificity was the largest at 148, which was taken as the minimum sample size required for the study. However, all 201 infants who completed the three assessments were included to enhance the precision of the estimates.

### Variables

Demographic information, including birthweight, gestational age, and neonatal risk factors, was extracted from the clinical database maintained at the Paediatric Neurology Unit, Teaching Hospital Karapitiya. Low birthweight was defined as less than 2500 g. Preterm birth was defined as a gestational age of less than 37 weeks.

The assessment protocol was integrated into the standard clinical follow‐up schedule. The initial GMA video to assess writhing movements was obtained shortly before hospital discharge, typically near term‐corrected gestational age. A second GMA video assessed fidgeting movements at 3 to 4 months after term. One HINE assessment was performed at approximately 5 to 6 months after term.

The GMA videos were made by a trained therapist using the standard Prechtl's protocol.^7^ These videos were then blindly assessed by three Prechtl‐certified raters. If there was a discrepancy in ratings, a fourth certified rater independently rated the videos to resolve such discrepancies. Given that such discrepancies occurred only in four cases, overall interrater agreement was estimated at 98% (*κ* = 0.98). All HINE assessments were performed by a team that included a paediatrician, an occupational therapist, and a physical therapist, all of whom were HINE‐trained. The team performed the HINE together and discussed any concerns to arrive at a consensus score.

At 24 months (range = 24.0–24.5 months), two consultant paediatric neurologists, who were blinded to the children's GMA and HINE scores or known concern for CP, independently performed a neurological examination to determine the presence or absence of CP. Interrater agreement was assessed based on the kappa statistic (*κ* = 0.94). Discrepancy was noted in only one case, which was resolved after further discussion between the neurologists, this being the first endpoint.^8^ Children diagnosed with CP were further classified according to the type of CP and level of disability using the Gross Motor Function Classification System (GMFCS).

At a median age of 30 months (interquartile range [IQR] = 27–34 months), children were assessed using the Bayley Scales of Infant and Toddler Development, Fourth Edition (Bayley‐4) by a certified assessor; their test protocols were scored using the Q‐Global digital platform (NCS Pearson, Minneapolis, MN, USA). The motor, cognitive, speech and language, and social–emotional domains of the Bayley‐4 were scored; children scoring 2 SDs below the mean or lower were classified as developmentally delayed based on standard population norms as provided by the Bayley‐4.^9^ A previous study using the Bayley‐3 showed that while there may be small differences between US and Sri Lankan children, the Bayley cognitive and motor subscales are reasonable to use in the assessment of infants in Sri Lanka.^10^ The assessment flow chart is summarized in Figure S1.

### Statistical analysis

The results of the GMA (fidgeting and writhing) were classified as follows. Writhing movements were scored into one of four categories: normal, poor repertoire, cramped synchronized, or chaotic. For our analysis, poor repertoire, cramped synchronized, and chaotic movements were categorized as abnormal. Likewise, fidgeting movements were classified as normal, abnormal, or absent. For the analysis, absent and abnormal movements were categorized as abnormal. The findings were cross‐tabulated against the presence or absence of CP based on the clinical diagnosis, which served as the criterion standard for classification. The diagnostic validity of the GMA was assessed based on sensitivity, specificity, positive predictive value, and negative predictive value. Sensitivity was defined as the proportion of individuals with a condition of interest who are correctly identified by a screening test as having said condition. Specificity was defined as the proportion of individuals without a condition who are correctly identified by a screening test as not having said condition. Positive predictive value was defined as the proportion of individuals with a positive screening test result who actually have the condition. Negative predictive value was defined as the proportion of individuals with a negative screening test result who do not have the condition.^11^

The HINE global score was used in the current analysis. The cut‐off score of the HINE for the detection of CP was determined using a receiver operating characteristic (ROC) analysis based on the Youden index (area under the curve [AUC] = 0.92, 95% confidence interval [CI] = 0.85–0.98, *p* < 0.001) due to unavailability of a cut‐off score to detect CP in the local context. The clinical diagnosis was used as the criterion standard to compute the threshold. Sensitivity and specificity at the selected thresholds from the ROC model were 79.7% and 89.5% respectively. Based on this analysis, a score of 58.5 was determined as the cut‐off value to categorize the HINE score as ‘normal’ or ‘abnormal’. The dichotomized HINE score was cross‐tabulated against the clinician's diagnosis of CP to calculate predictive measures for the assessment of its diagnostic validity. Likewise, the ROC analysis was used to generate non‐CP‐related developmental delay using the Bayley‐4 and clinical diagnosis as the criterion standard. The threshold cut‐off scores were selected using the Youden index, which was 58.5 for all aspects of non‐CP‐related developmental delay; non‐CP‐related motor delay (AUC = 0.84; 95% CI = 0.77–0.92, *p* < 0.001); social–emotional delay (AUC = 0.73, 95% CI = 0.62–0.84, *p* < 0.001); cognitive delay (AUC = 0.76, 95% CI = 0.67–0.86, *p* < 0.001); and speech and language delay (AUC = 0.64, 95% CI = 0.56–0.73, *p* = 0.002). Therefore, a HINE score less than 58.5 was considered abnormal in all aspects of the assessment. The sensitivity and specificity at the selected thresholds from the ROC model were 86.2% and 65.2% for non‐CP‐related motor delay, 85.0% and 47.9% for social–emotional delay, 86.1% and 51.6% for cognitive delay, and 86.8% and 34.4% for speech and language delay respectively.

The dichotomized GMA (writhing/fidgeting) findings and HINE scores were cross‐tabulated against the presence or absence of the aspects of developmental delay. Predictive measures (sensitivity, specificity, positive predictive value, and negative predictive value) of single assessments, as well as combinations of these assessments, were calculated with a 95% CI. The purpose of this analysis was to test whether combining the GMA and HINE assessments improved the identification of cases compared to using them singly. In the combined assessment, if at least one assessment (writhing, fidgeting, or HINE) was abnormal, it was categorized as abnormal. If all three assessments were normal, it was categorized as normal.

SPSS v25 (IBM Corp., Armonk, NY, USA) was used to calculate the cell counts for cross‐tabulation. Predictive measures were calculated using the diagnostic test evaluation calculator freely available in the MedCalc statistical software (MedCalc Software Ltd., Ostend, Belgium). The alpha level of significance was set at 0.05.

### Ethical considerations

The protocols used in the study were approved by the Ethics Review Committee, Faculty of Medicine, University of Ruhuna (no. 2021.P.081); written informed consent was obtained from all primary caregivers of the participating children before data collection.

## RESULTS

A total of 416 infants enrolled in the study; 201 (48.3% of total, 128 males, 73 females) completed all three assessments and their data was used for the analysis. There was no significant difference (*p* = 0.384) in the rate of CP in the 215 children who did not complete all three assessments (CP rate = 26/215 [12.1%]) compared to the 201 individuals who did complete all three assessments (CP rate = 19/201 [9.5%]). Furthermore, the prevalence of risk factors was similar in the two groups (Table 1).

Most participants were Sinhalese (96.5%) and male (63.7%). Half of the participants had been delivered by emergency caesarean section (50.7%) followed by normal vaginal delivery (39.3%), elective caesarean section (6.5%), and assisted vaginal delivery (3.5%). The most common risk factors identified were low birthweight (55.7%), preterm birth (52.2%), needing artificial ventilation at birth (22.9%), and requiring neonatal resuscitation (25.9%) (Table 1). In addition, central nervous system infections occurred in 13.9% of the cohort and congenital infections in 3.0%.

**FIGURE 1:**
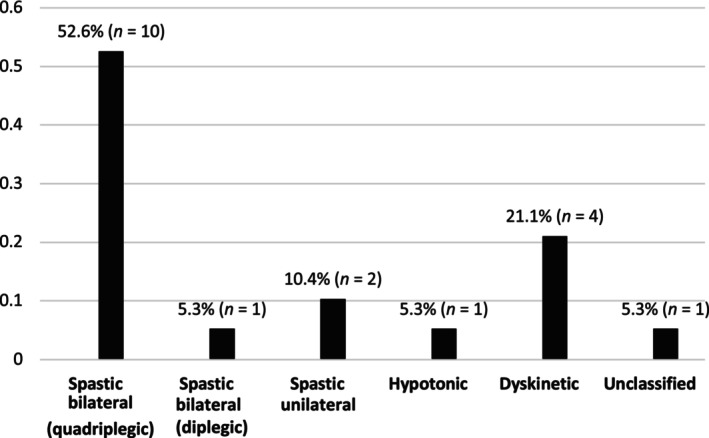
Cerebral palsy subtypes (*n* = 19). Note that the Surveillance of Cerebral Palsy in Europe classification system has not been used; rather, subtypes of cerebral palsy have been classified as per Rosenbaum et al.^8^ Y‐axis: percent of total cohort.

Nineteen children (9.5%) were diagnosed with CP at 24 months of age, most (52.6%) of whom had spastic bilateral (quadriplegic) CP (Figure 1). The degree of motor delay for children with CP was determined using the GMFCS and included children classified in GMFCS level I (10.5%), GMFCS level II (10.5%), GMFCS level IV (47.4%), and GMFCS level V (31.3%). There were 182 children without CP, two of whom were lost to follow‐up.

Of the remaining 180 children, all were assessed with the Bayley‐4. Based on the Bayley‐4, 35.0% of the group without CP had at least one type of developmental delay that included speech and language (33.3%), cognitive (16.7%), social–emotional (16.7%), and motor (12.8%) delay. The domain of adaptive behaviour relies entirely on parent impression and was felt to be inaccurate by the study team. Therefore, it was not included in the outcome analyses.

### Early detection of CP among high‐risk infants

All children in the study cohort underwent assessment of writhing before hospital discharge, assessment of fidgeting (median age = 17 weeks, IQR = 14–21 weeks), and the HINE (median age = 27 weeks, IQR = 21–31 weeks). Among them, 107 (53.2%) had abnormal writhing movements, 21 (10.4%) had abnormal fidgeting movements, and 54 (26.8%) had an abnormal HINE. As Table 2 shows, of the three assessments taken individually (GMA for writhing movements, GMA for fidgeting movements, HINE), the GMA for fidgeting movements provided the highest sensitivity and specificity for the early detection of CP. When the GMA and HINE scores were combined, the sensitivity of the tests for the detection of CP increased up to 100%; other combinations also had high sensitivity, enabling identification of CP in vulnerable infants (Table 3).

**TABLE 2:** Diagnostic validity with 95% confidence intervals of the General Movements Assessment and Hammersmith Infant Neurological Examination for the detection of cerebral palsy (*n* = 201).

		Writhing, % (95% CI)	Fidgeting, % (95% CI)	HINE, % (95% CI)
Group born preterm (*n* = 105)	Sensitivity	100.0 (63.1–100.0)	100.0 (63.1–100.0)	87.5 (47.4–99.7)
	Specificity	55.7 (45.2–65.7)	97.9 (92.8–99.8)	80.4 (71.1–87.8)
	PPV	15.7 (12.9–18.8)	80.0 (50.4–94.0)	26.9 (18.5–37.3)
	NPV	100.0 (93.4–100.0)	100.0 (96.2–100.0)	98.7 (92.6–99.8)
Group born at term (*n* = 96)	Sensitivity	81.8 (48.2–97.7)	99.9 (58.7–99.8)	90.9 (58.7–99.8)
	Specificity	44.7 (33.9–55.9)	98.8 (93.6–99.6)	78.8 (68.6–86.9)
	PPV	16.1 (12.0–21.2)	90.9 (58.6–98.6)	35.7 (26.1–46.6)
	NPV	95.0 (84.1–98.6)	98.8 (92.8–99.8)	98.5 (91.2–99.8)
All (*n* = 201)	Sensitivity	89.5 (66.9–98.7)	94.7 (73.9–99.9)	89.5 (66.9–98.7)
	Specificity	50.6 (43.1–58.0)	98.4 (95.3–99.7)	79.7 (73.1–85.3)
	PPV	15.9 (13.2–18.9)	85.7 (66.0–94.9)	31.5 (24.9–38.9)
	NPV	97.9 (92.5–99.4)	99.4 (96.4–99.9)	98.6 (95.1–99.6)

Abbreviations: CI, confidence interval; HINE, Hammersmith Infant Neurological Examination; NPV, negative predictive value; PPV, positive predictive value.

**TABLE 3:** Diagnostic validity with 95% confidence intervals of the combined methods used to detect cerebral palsy in the study cohort (*n* = 201).

		GMA (writhing + fidgeting), % (95% CI)	Writhing + HINE, % (95% CI)	Fidgeting + HINE, % (95% CI)	Modified approach (GMA + HINE), % (95% CI)
Group born preterm (*n* = 105)	Sensitivity	100.0 (63.1–100.0)	100.0 (63.1–100.0)	100.0 (63.1–100.0)	100.0 (63.1–100.0)
	Specificity	54.6 (44.2–66.8)	45.4 (35.2–55.8)	79.4 (69.9–86.9)	44.3 (34.2–54.7)
	PPV	15.4 (12.8–18.5)	13.1 (11.2–15.3)	28.6 (21.3–37.1)	12.9 (11.0–15.0)
	NPV	100.0 (93.3–100.0)	100.0 (91.9–100.0)	100.0 (95.3–100.0)	100.0 (91.8–100.0)
Group born at term (*n* = 96)	Sensitivity	100.0 (75.5–100.0)	90.9 (58.7–99.7)	100.0 (75.5–100.0)	100.0 (75.1–100.0)
	Specificity	43.5 (32.8–54.7)	36.5 (26.3–47.6)	77.7 (67.3–85.9)	35.3 (25.2–46.4)
	PPV	18.6 (15.9–21.6)	15.6 (12.6–19.2)	36.7 (28.0–46.3)	16.7 (14.6–18.9)
	NPV	100.0 (90.5–100.0)	96.8 (82.4–99.5)	100.0 (94.6–100.0)	100.0 (88.4–100.0)
All (*n* = 201)	Sensitivity	100.0 (82.4–100.0)	94.7 (73.9–99.9)	100.0 (82.4–100.0)	100.0 (82.4–100.0)
	Specificity	49.5 (41.9–56.9)	41.2 (33.9–48.7)	78.6 (71.9–84.3)	40.1 (32.9–46.7)
	PPV	17.1 (15.2–19.3)	14.4 (12.5–16.5)	32.8 (26.9–39.2)	14.8 (13.4–16.4)
	NPV	100.0 (95.9–100.0)	98.7 (91.7–99.8)	100.0 (97.5–100.0)	100.0 (95.1–100.0)

Abbreviations: CI, confidence interval; GMA, General Movements Assessment; HINE, Hammersmith Infant Neurological Examination; NPV, negative predictive value; PPV, positive predictive value.

### Early detection of non‐CP‐related developmental delay among high‐risk infants

Individually, the GMA and HINE had low predictive value for non‐CP‐related developmental delay (Table 4), although when combined, sensitivity for predicting motor (86.9%), cognitive (86.7%), and social–emotional (83.3%) delay was reasonable; however, specificity was low for all three (43.6%, 45.3%, and 44.7% respectively; Table 5).

**TABLE 4:** Diagnostic validity with confidence intervals (95%) of General Movements Assessment (writhing and fidgety) and Hammersmith Infant Neurological Examination (HINE) for detection of non‐cerebral palsy development delays (*n* = 201).

Non‐CP‐related developmental delay	Method	Sensitivity, % (95% CI)	Specificity, % (95% CI)	PPV, % (95% CI)	NPV, % (95% CI)
Motor	Writhing	60.9 (38.5–80.3)	52.2 (44.1–60.3)	15.7 (11.5–21.2)	90.1 (84.3–93.9)
	Fidgety	8.7 (1.1–28.0)	99.4 (96.5–99.9)	66.7 (15.9–95.5)	88.1 (86.7–89.4)
	HINE	65.2 (42.7–83.6)	85.9 (79.6–91.0)	40.5 (29.5–52.7)	94.4 (90.6–96.7)
Cognitive	Writhing	63.3 (43.9–80.1)	53.3 (45.0–61.5)	21.4 (16.4–27.2)	87.9 (81.6–92.3)
	Fidgety	10.0 (2.1–26.5)	100.0 (97.6–100.0)	100.0 (29.2–100.0)	84.8 (83.1–86.2)
	HINE	53.3 (34.3–71.7)	86.0 (79.4–91.1)	43.2 (31.2–56.2)	90.2 (86.2–93.1)
Social–emotional	Writhing	60.0 (40.6–77.34)	52.7 (44.4–60.9)	20.2 (15.3–26.2)	86.8 (80.6–91.3)
	Fidgety	10.0 (2.1–26.5)	100.0 (97.6–100.0)	100.0 (29.2–100.0)	84.8 (83.1–86.2)
	HINE	50.0 (31.3–68.7)	85.3 (78.6–90.6)	40.5 (28.7–53.6)	89.5 (85.6–92.5)
Language and speech	Writhing	51.7 (38.4–64.8)	51.7 (42.4–60.9)	34.8 (28.2–42.1)	68.1 (60.9–74.5)
	Fidgety	5.0 (1.0–13.9)	100.0 (96.9–100.0)	100.0 (29.2–100.0)	67.8 (66.5–69.1)
	HINE	35.0 (23.1–48.4)	86.7 (79.3–92.2)	56.7 (42.6–69.9)	72.7 (68.6–76.5)

Abbreviations: CI, confidence interval; CP, cerebral palsy; HINE, Hammersmith Infant Neurological Examination; NPV, negative predictive value; PPV, positive predictive value.

**TABLE 5:** Diagnostic validity with 95% confidence intervals of the combined methods used to detect non‐cerebral‐palsy‐related developmental delay (*n* = 201).

Domain	Method	Sensitivity, % (95% CI)	Specificity, % (95% CI)	PPV, % (95% CI)	NPV, % (95% CI)
Motor	GMA (fidgeting + writhing)	65.2 (42.7–83.6)	51.6 (43.5–59.6)	16.5 (12.3–21.7)	91.0 (85.0–94.8)
	Fidgeting + HINE	69.6 (47.1–86.8)	85.4 (78.8–90.5)	41.0 (30.4–52.5)	95.0 (91.1–97.3)
	Writhing + HINE	82.6 (61.2–95.1)	44.6 (36.7–52.7)	17.9 (14.7–21.6)	94.6 (87.6–97.8)
	Modified approach (GMA + HINE)	86.9 (66.4–97.2)	43.6 (35.7–51.8)	18.5 (15.6–21.9)	95.8 (88.6–98.5)
Cognitive	Fidgeting + writhing	70.0 (50.6–85.3)	53.3 (45.0–61.5)	23.1 (18.3–28.6)	89.9 (83.5–94.0)
	Fidgeting + HINE	60.0 (40.6–77.3)	86.0 (79.4–91.1)	46.2 (34.4–58.4)	91.5 (87.4–94.4)
	Writhing + HINE	80.0 (61.4–92.3)	45.3 (37.2–53.7)	22.6 (18.9–26.9)	91.9 (84.4–95.6)
	Modified approach (GMA + HINE)	86.7 (69.3–96.2)	45.3 (37.2–53.7)	24.1 (20.6–27.9)	94.4 (87.0–97.7)
Social–emotional	GMA (fidgeting + writhing)	66.7 (47.2–82.7)	52.7 (44.4–60.9)	21.9 (17.2–27.6)	88.8 (82.3–93.1)
	Fidgeting + HINE	56.7 (37.4–74.5)	85.3 (78.6–90.6)	43.6 (31.9–55.9)	90.8 (86.7–93.7)
	Writhing + HINE	76.7 (57.7–90.1)	44.7 (36.6–52.9)	21.7 (17.8–26.1)	90.5 (83.0–94.9)
	Modified approach (GMA + HINE)	83.3 (65.3–94.4)	44.7 (36.6–52.9)	23.2 (19.5–27.2)	93.1 (85.5–96.8)
Language and speech	GMA (fidgeting + writhing)	55.0 (41.6–67.8)	51.7 (42.4–60.9)	32.3 (29.8–43.3)	69.7 (62.3–76.1)
	Fidgeting + HINE	38.3 (26.1–51.8)	86.7 (79.3–92.2)	58.9 (45.1–71.5)	73.8 (69.5–77.6)
	Writhing + HINE	65.0 (51.6–76.9)	44.2 (35.1–53.5)	36.8 (31.3–42.6)	71.6 (62.9–79.0)
	Modified approach (GMA + HINE)	68.3 (55.0–79.7)	44.2 (35.1–53.3)	37.9 (32.6–43.6)	73.6 (64.6–80.9)

Abbreviations: CI, confidence interval; GMA, General Movements Assessment; HINE, Hammersmith Infant Neurological Examination; NPV, negative predictive value; PPV, positive predictive value.

## DISCUSSION

We report, to our knowledge, the first single‐site, prospective study using a combination of the HINE and GMA to predict CP and other developmental delays in children born in an LMIC. Our main finding is that even without data from advanced neuroimaging such as brain MRI, as is often done in developed nations, it is possible to predict which high‐risk infants will develop CP with almost 100% sensitivity, predict with 86% sensitivity children who will experience cognitive delay, and predict with 83% sensitivity children who will experience social–emotional delay. While specificity is lower, these findings suggest that very few children who develop CP or cognitive delay will be missed by this approach of integrating the GMA and HINE into the routine care of children at risk, even without MRI. This approach is feasible in resource‐limited settings, allowing providers to target early developmental therapies to those children who will benefit most from these services.

Preclinical^12^ and clinical^13^, ^14^ work supports the benefit of beginning developmental therapy as soon as possible in children with CP. Therefore, early diagnosis is essential to target developmental therapy appropriately and maximize developmental outcome. Unfortunately, early diagnosis can be challenging, particularly in LMICs where access to specialty services and advanced neuroimaging is limited.^15^

Several assessments have been evaluated for accuracy in predicting CP and other developmental problems in children born preterm. Brain MRI is predictive of CP;^16^ however, it is not always available in resource‐limited settings. The GMA assesses the quality of an infant's spontaneous movements over the first 5 months of life.^7^, ^17^ Studies primarily from HICs showed that the GMA is predictive of CP and other developmental delays at 24 months of age, particularly if the GMA is performed during the fidgeting period^14^, ^18^, ^19^ or if it is repeated over time.^17^, ^20^, ^21^ The HINE is another clinical tool that can predict CP, with higher predictive power when done later, at 9 or 12 months of age.^22^ Current recommendations are to diagnose CP in children under 5 months (corrected for gestational age) using a combination of brain MRI and either the GMA or HINE. In children over 5 months of age, brain MRI and HINE are recommended for diagnosis. In countries with limited access to MRI, the HINE is suggested for diagnosis.^3^

A common clinical practice in Sri Lanka, as in many LMICs, is to hospitalize children after preterm delivery until they are stable, often close to term‐corrected age, followed by routine outpatient follow‐up appointments every 2 to 3 months for vaccinations and well‐child care. This routine clinical schedule was followed in the current study: the first GMA (writhing) was performed before hospital discharge, with the second GMA (fidgeting) performed at a corrected age of 3 to 4 months during a routine follow‐up visit. This minimized participant burden but still provided an assessment of writhing (GMA before 44 weeks gestational age) and fidgeting movements (GMA at 3–4 months corrected age). Finally, the HINE was performed at a corrected age of 5 to 6 months. Thus, the current study's protocol minimized burden on families and the health care system, which is an important issue in countries such as Sri Lanka where many families live in poverty (currently approximately 30%)^23^ and access to specialists, including paediatric neurologists, is limited.

The current study contributes to the limited early detection data from LMICs,^24^, ^25^, ^26^, ^27^, ^28^ where resources and risk factors differ from HICs.^29^ As is often seen in LMICs, our cohort had a higher incidence of birth asphyxia and perinatal infection, and two times as much spastic bilateral (quadriplegic) CP, than in HICs.^30^ These differences in risk factors and types of CP may influence early identification strategies; thus, it is important that studies from LMICs be reported to reflect real‐world experience in developing countries, which is where most children with CP and other developmental disabilities live.

Using the Bayley‐4 we were able to extend our findings to motor and cognitive outcomes in multiple domains. This included an assessment of cognitive and social–emotional functioning, to our knowledge a finding not previously reported. We found that the combination of GMA and HINE was sensitive to cognitive (86.7%) and social–emotional (83%) outcomes, but with low specificity (45.3% and 44.7% respectively). Thus, our modified schedule of using the GMA and HINE should identify most children who will experience cognitive and social–emotional delay, albeit with a high number of false positives. From a practical standpoint, this is acceptable. In a country such as Sri Lanka, with free health care and access to developmental therapists, it is better to provide developmental therapy and education to too many children and their families, than to risk missing an opportunity to provide these services to those children who will benefit. Therefore, our modified schedule of the GMA and HINE has value in the early detection of both CP and cognitive delay.

A limitation of this study is that only approximately 50% of the participants who enrolled completed all three study assessments. While this could reflect retention bias, unique events occurring in Sri Lanka during the study period influenced retention (COVID‐19 pandemic, terrorist bombings of Easter 2019, and economic collapse causing severe fuel shortages). Also, we did not include the adaptive domains of the Bayley‐4 in our analysis because these data were outliers, possibly because they are based on caregiver questionnaires and not objective assessment, which our assessors felt was unreliable (related to cultural factors inhibiting honest answers). In addition, this study used the HINE and GMA, which were originally designed and validated in HICs, not LMICs. This is an important issue because typically developing low‐risk infants from some LMICs may score lower on a standard neurological examination than infants from HICs.^31^ However, the HINE and GMA were deemed appropriate in Sri Lanka because Sri Lankan infants and toddlers have been found to perform at comparable levels to children in the USA on the motor and cognitive Bayley domains.^10^ An additional potential limitation is that we used our study cohort for ROC modelling and determination of CP and non‐CP thresholds; this was done because these data are not available for non‐CP‐related developmental delays, or for determining CP in children in our geographical region of the world. Finally, a weakness of this study is that it was conducted at a single site with a cohort that was exclusively Sinhalese. Future studies will include other sites in Sri Lanka to access the multicultural richness of the country, including the Tamil and Muslim populations.

### Conclusion

This study shows that in a developing nation with limited resources, integrating the GMA and HINE into routine clinical care minimizes burden on families. This strategy is both feasible and extremely sensitive in predicting CP, and to a lesser extent in predicting cognitive and social–emotional delay. While high sensitivity with lower specificity overidentifies children who will experience developmental problems, this is reasonable from a public health perspective in a developing nation such as Sri Lanka, where an adequate number of developmental therapists are available to treat these children.

## Supporting information


**Figure S1:** Assessment flow chart.

## Data Availability

The data that support the findings of this study are available from the corresponding author upon reasonable request.

## References

[1] Hadders‐Algra M. Early diagnostics and early intervention in neurodevelopmental disorders—age‐dependent challenges and opportunities. J Journal of clinical medicine. 2021;10(4):861. 10.3390/jcm10040861 PMC7922888 33669727

[2] Marschik PB , Pokorny FB , Peharz R , Zhang D , O'Muircheartaigh J , Roeyers H , et al. A novel way to measure and predict development: A heuristic approach to facilitate the early detection of neurodevelopmental disorders. J Current neurology neuroscience reports. 2017;17:1–15. 10.1007/s11910-017-0748-8 PMC5384955 28390033

[3] Dubowitz LM , Dubowitz V , Mercuri E . The neurological assessment of the preterm and full‐term newborn infant. Cambridge University Press; 1999 Jan 26 AND Dubowitz L, Mercuri E, Dubowitz V. An optimality score for the neurologic examination of the term newborn. The Journal of pediatrics. 1998 Sep 1;133(3):406–16). 9738726 10.1016/s0022-3476(98)70279-3

[4] Novak I , Morgan C , Adde L , Blackman J , Boyd RN , Brunstrom‐Hernandez J , et al. Early, accurate diagnosis and early intervention in cerebral palsy: advances in diagnosis and treatment. J JAMA pediatrics. 2017;171(9):897–907. 10.1001/jamapediatrics.2017.1689 PMC9641643 28715518

[5] Libertus K , Hauf P . Motor skills and their foundational role for perceptual, social, and cognitive development. Frontiers in psychology. 2017 Mar 6;8. 10.3389/fpsyg.2017.00301 PMC5337521 28321199

[6] Hajian‐Tilaki K. Sample Size Estimation in Diagnostic Test Studies of Biomedical Informatics. Journal of biomedical informatics. 2014;48. 10.1016/j.jbi.2014.02.013 24582925

[7] Ferrari F , Einspieler C , Prechtl H , Bos A , Cioni G . Prechtl's method on the qualitative assessment of general movements in preterm, term and young infants: Mac Keith Press; 2004 10.1016/s0378-3782(97)00092-3 9467693

[8] Rosenbaum P , Paneth N , Leviton A , Goldstein M , Bax M , Damiano D , Dan B , Jacobsson B . A report: the definition and classification of cerebral palsy April 2006. Dev Med Child Neurol Suppl. 2007 Feb 1;109(suppl 109):8–14. 17370477

[9] Bayley N , Aylward GP . Bayley scales of infant and toddler development fourth edition (Bayley‐4). Bloomington, MN: NCS Pearson. 2019.

[10] Godamunne P , Liyanage C , Wimaladharmasooriya N , Pathmeswaran A , Wickremasinghe AR , Patterson C , Sathiakumar N . Comparison of performance of Sri Lankan and US children on cognitive and motor scales of the Bayley scales of infant development. BMC research notes. 2014 Dec;7:1–5. 24886547 10.1186/1756-0500-7-300 PMC4025536

[11] Trevethan R. Sensitivity, Specificity, and Predictive Values: Foundations, Pliabilities, and Pitfalls in Research and Practice. Frontiers in Public Health 2017 Nov; 5:307 10.3389/fpubh.2017.00307 PMC5701930 29209603

[12] Martin JH , Chakrabarty S , Friel KM . Harnessing activity‐dependent plasticity to repair the damaged corticospinal tract in an animal model of cerebral palsy. Developmental Medicine & Child Neurology. 2011 Sep;53:9–13 10.1111/j.1469-8749.2011.04055.x PMC3187875 21950387

[13] Nordhov S , Ronning J , Ulvund S , Dahl L , Kaaresen P . Early Intervention Improves Behavioral Outcomes for Preterm Infants: Randomized Controlled Trial. Pediatrics. 2011;129:e9–e16. 22184645 10.1542/peds.2011-0248

[14] Spittle A , Orton J , Anderson P , Boyd R , Doyle L . Early developmental intervention programs post hospital discharge to prevent motor and cognitive impairments in preterm infants. Cochrane database of systematic reviews (Online). 2012;12:CD005495. 10.1002/14651858.CD005495.pub3 23235624

[15] Bitta M , Kariuki SM , Abubakar A , Newton CRJWor. Burden of neurodevelopmental disorders in low and middle‐income countries: A systematic review and meta‐analysis. 2017;2 10.12688/wellcomeopenres.13540.3 PMC5964629 29881784

[16] Bosanquet M , Copeland L , Ware R , Boyd R . A systematic review of tests to predict cerebral palsy in young children. Developmental medicine and child neurology. 2013;55:418–26. 23574478 10.1111/dmcn.12140

[17] Prechtl HF . State of the art of a new functional assessment of the young nervous system. An early predictor of cerebral palsy. J Early human development. 1997;50(1):1–11. 10.1016/s0378-3782(97)00088-1 9467689

[18] Guzzetta A , Belmonti V , Battini R , Boldrini A , Paolicelli PB , Cioni G . Does the assessment of general movements without video observation reliably predict neurological outcome? European Journal of Paediatric Neurology. 2007 Nov 1;11(6):362–7. 17428706 10.1016/j.ejpn.2007.03.003

[19] Hadders‐Algra M. General Movements: A Window for Early Identification of Children at High Risk for Developmental Disorders. The Journal of pediatrics. 2004;145:S12–8. 15292882 10.1016/j.jpeds.2004.05.017

[20] Akcakaya N , Altunalan T , Doğan T , Yılmaz A , Yapici Z . Correlation of Prechtl Qualitative Assessment of General Movement Analysis with Neurological Evaluation: The Importance of Inspection in Infants. Turk Noroloji Dergisi. 2019:63–70.

[21] Einspieler C , Prechtl HFR , Bos A , Ferrari F , Cioni G . Prechtl's method on the qualitative assessment of general movements in preterm, term and young infants. Clin Dev Med. 2004;167:1–91. 10.1016/s0378-3782(97)00092-3 9467693

[22] Romeo DM , Ricci D , Brogna C , Mercuri EJDM . Use of the Hammersmith Infant Neurological Examination in infants with cerebral palsy: a critical review of the literature. J Developmental Medicine Child Neurology. 2016;58(3):240–5. 10.1111/dmcn.12876 26306473

[23] Valavanidis A. World Bank Report: Extreme Poverty is Rising Again. From 660 million people (8.5% world population) in 2019 to 733 million in 2022. 2023;1:1–23.

[24] Manacero S , Marschik P , Nunes M , Einspieler C . Is it possible to predict the infant's neurodevelopmental outcome at 14 months of age by means of a single preterm assessment of General Movements? Early human development. 2011;88:39–43. 21775078 10.1016/j.earlhumdev.2011.06.013 PMC3253387

[25] Dimitrijević L , Bjelakovic B , Colovic H , Mikov A , Vesna Z , Kocic M , et al. Assessment of general movements and heart rate variability in prediction of neurodevelopmental outcome in preterm infants. Early human development. 2016;99:7–12. 27372636 10.1016/j.earlhumdev.2016.05.014

[26] Aker K , Thomas N , Adde L , Koshy B , Martinez‐Biarge M , Nakken I , Padankatti CS , Støen R . Prediction of outcome from MRI and general movements assessment after hypoxic‐ischaemic encephalopathy in low‐income and middle‐income countries: data from a randomised controlled trial. Archives of Disease in Childhood‐Fetal and Neonatal Edition. 2022 Jan 1;107(1):32–8. 34112719 10.1136/archdischild-2020-321309 PMC8685634

[27] Kadam AS , Nayyar SA , Kadam SS , Patni BC , Khole MC , Pandit AN , Kabra NS . General Movement Assessment in Babies Born Preterm: Motor Optimality Score–Revised (MOS‐R), Trajectory, and Neurodevelopmental Outcomes at 1 Year. The Journal of Pediatrics: X. 2023 Mar 1;8: 100084. 37334030 10.1016/j.ympdx.2022.100084 PMC10236536

[28] Zhussupova Z , Jaxybayeva A , Ayaganov D , Tekebayeva L , Mamedbayli A , Tamadon A , Zharmakhanova G . General movement assessment efficacy for assessment of nervous system integrity in children after hypoxic‐ischemic encephalopathy in middle income countries. Early Human Development. 2024 Mar 24; 192: 105992. 38574696 10.1016/j.earlhumdev.2024.105992

[29] Jahan I , Muhit M , Hardianto D , Laryea F , Chhetri AB , Smithers‐Sheedy H , McIntyre S , Badawi N , Khandaker G . Epidemiology of cerebral palsy in low‐and middle‐income countries: preliminary findings from an international multi‐centre cerebral palsy register. Developmental Medicine & Child Neurology. 2021 Nov;63(11):1327–36. 34031872 10.1111/dmcn.14926

[30] Smithers‐Sheedy H , McIntyre S , Gibson C , Meehan E , Scott H , Goldsmith S , et al. A special supplement: Findings from the Australian Cerebral Palsy Register, birth years 1993 to 2006. Developmental Medicine and Child Neurology. 2016;58(Suppl 2): 5–10. 10.1111/dmcn.13026 26762930

[31] Lawford HL , Nuamah MA , Liley HG , Lee AC , Kumar S , Adjei AA , Bora S , Samba A , Badoe EV , Botchway F , Gyasi RK . Neonatal neurological examination in a resource‐limited setting: What defines normal? European journal of paediatric neurology. 2020 Nov 1;29:71–80. 10.1016/j.ejpn.2020.08.010 33036879

