# Supplementary figures and images for: Prediction of cerebral palsy and cognitive delay among high‐risk children in a developing nation: A successful early detection programme
Source: Dev Med Child Neurol. 2024 Dec 28;67(7):892–900. doi: 10.1111/dmcn.16197 (PMC12134449; doi:10.1111/dmcn.16197)

**Figure 1: Assessment Flow Chart**

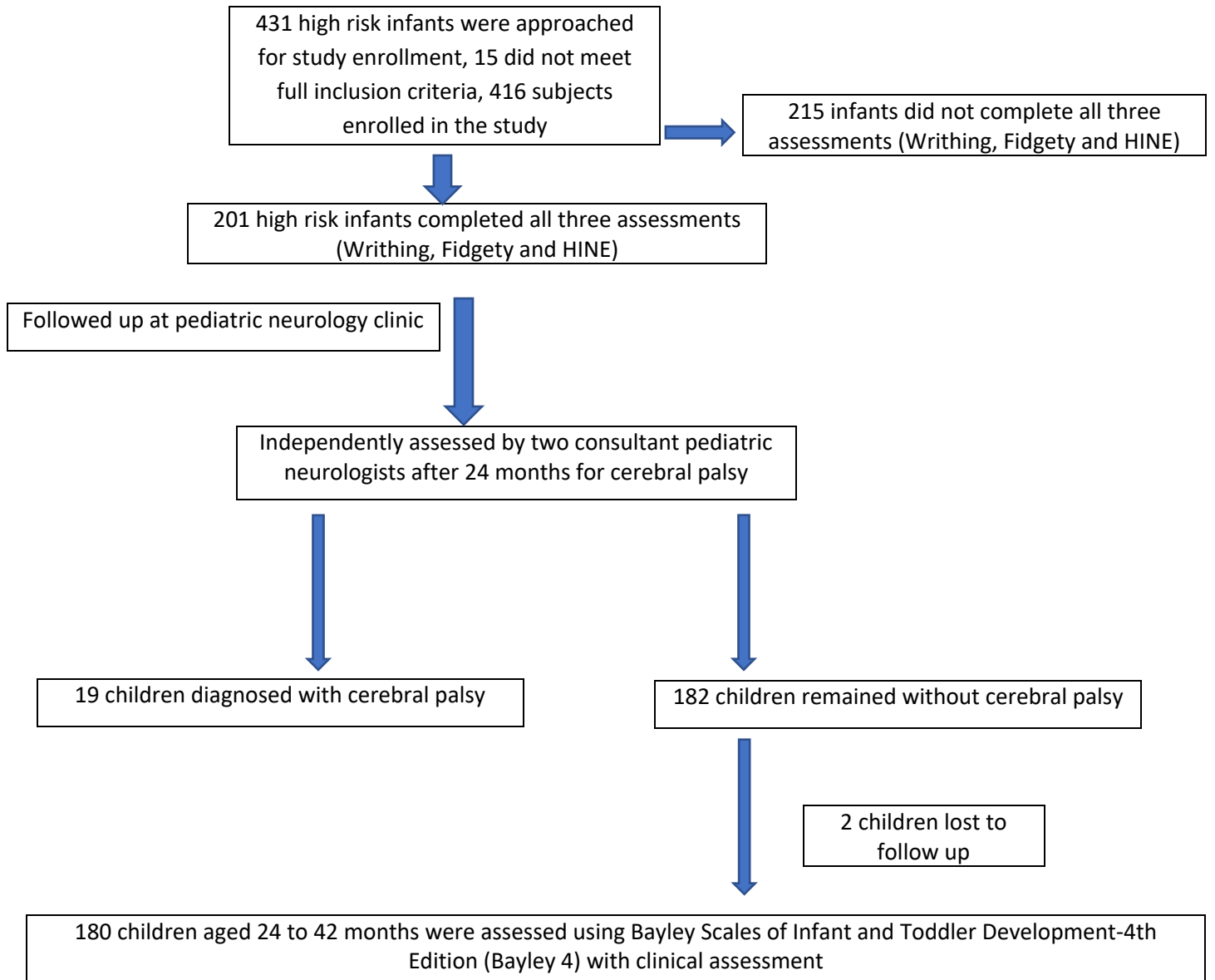

Supplement: Supplementary file 1 — Figure S1: Assessment flow chart. [file DMCN-67-892-s001.pdf]
